# Uterine Prolapse: From Antiquity to Today

**DOI:** 10.1155/2012/649459

**Published:** 2011-11-14

**Authors:** Keith T. Downing

**Affiliations:** Division of Female Pelvic Medicine and Reconstructive Surgery, Department of Obstetrics & Gynecology and Women's Health, Albert Einstein College of Medicine/Montefiore Medical Center, Bronx, NY 10461, USA

## Abstract

Uterine prolapse is a condition that has likely affected women for all of time as it is documented in the oldest medical literature. By looking at the watershed moments in its recorded history we are able to appreciate the evolution of urogynecology and to gain perspective on the challenges faced by today's female pelvic medicine and reconstructive surgeons in their attempts to treat uterine and vaginal vault prolapse.

*“He who cannot render an account to himself of at least three thousand years of time, will always grope in the darkness of inexperience”*

—Goethe, Translation of Panebaker

## 1. Introduction

This special issue provides urogynecologists with the opportunity to explore recent advances that have and will continue to propel our subspecialty forward. Simultaneously, it provides us with the opportunity to look back and appreciate the landmark moments that have led us to our current state of affairs. It is with this spirit, mindful of Goethe's words, that this paper will focus its attention on a brief history of the management of uterine prolapse. 

## 2. Antiquity to the Common Era

Uterine prolapse is an ailment that has seemingly affected women for all of time. In fact, the problem of uterine prolapse and its potential treatment is described in the oldest documented medical literature, the Egyptian Papyri, where it is written, “of a woman whose posterior, belly, and branching of her thighs are painful, say thou as to it, it is the falling of the womb,” (Kahun papyrus ca. 1835 B.C.E.) [1]. The Ebers papyrus goes on to recommend “to correct a displaced womb: with oil of earth (petroleum) with fedder (manure) and honey; rub the body of the patient,” (Ebers papyrus ca. 1550 B.C.E.) [2].

Over one thousand years later, during the time of Hippocrates (c. 460–377 B.C.E.) and the subsequent generations that he influenced, the prevailing medical thought was that the uterus acted as an animal unto itself. This concept led to treatments such as fumigation, in which pleasant fumes would be placed at a woman's head and vile ones near her prolapsed womb, in order to stimulate the uterus to retreat. Polybus, a pupil of Hippocrates (and his son-in-law), wrote in his noted text “On Diseases of Women,” of other therapies for uterine prolapse including the application of an astringent to the womb followed by placement of a vinegar soaked sponge, or halved pomegranate. If these measures failed, women were subjected to succussion—the practice of tying a woman upside down by her feet to a fixed frame and bouncing her repeatedly until her prolapse reduced then leaving her bed bound for three days with her legs tied together [3]. 

However, a gradual shift in medical thought began to occur toward the end of the Hippocratic era. Medicine slowly began to free itself from the influence of theurgy. By the first century C.E. Soranus, the most notable gynecologist of antiquity, would rebuke the Hippocratic approaches to treating uterine prolapse. He considered fumigation nonsensical, regarded the use of pomegranates as bruising, and deemed succussion unbearable. Instead, in his monumental treatise, “Gynecology,” Soranus prescribed the following: “… bathe the prolapsed part of the uterus with much lukewarm olive oil, and make a woolen tampon corresponding in shape and diameter to the vagina and wrap it in very thin clean linen… one should dip it briefly in vinegar… acacia juice… or wine, and apply it to the uterus and move the whole prolapsed part, forcing it up gently until the uterus has reverted to its proper place and the whole mass of wool is in the vagina” [4]. Yet, despite this therapeutic advance, outdated notions about the uterus would persist. As late as the second century C.E., prominent Greek physician Aretaeus the Cappadocian, in his “Causes and Indications of Acute and Chronic Diseases,” still described the uterus as, “an animal within an animal” [5].

Despite Soranus's vast knowledge of obstetrics and gynecology, female pelvic anatomy remained poorly understood. Physicians of the age commonly referred to the uterus as mater (Latin for mother) or hystera (Greek for womb) in the plural form, believing the uterus consisted of more than one chamber [3]. Had it not been for Rome's prohibition on the use of human cadavers, this belief might have been dispelled by the work of Galen, the Rome based physician and anatomist. However, Galen was left to extrapolate his understanding of human anatomy from dissections and vivisections of lower animals in which the finding of uterine horns was commonplace [3].

The Mediaeval era brought about a return to theurgy, and medicine, including the management of uterine prolapse, regressed. It was during the Middle Ages that fantastical concepts regarding female pelvic anatomy emerged. The seven cells doctrine was one such concept. It stated that the uterus consisted of seven compartments, three on each side and one in the middle and posited that female fetuses developed on the left, male fetuses on the right, hermaphrodites in the middle [6]. Beliefs from the Hippocratic era resurfaced and as late as 1603, a text by Roderigo de Castro advised that the prolapsed uterus, “be attacked with a red-hot iron as if to burn, whereupon fright will force the prolapsed part to recede into the vagina” [3]. While the practice of medicine during the Middle Ages left much to be desired, in the middle of the fifteenth century changes in the way people thought about art and philosophy would soon lead to new ways of thinking in medicine.

The Renaissance grew out of Florence where a collection of artists and intellectuals began to focus on the works and ways of the classical age. This led to a renewed attention to the beauty of nature, including the human form [7]. Artists took part in private anatomic dissections to advance their training, something physicians of that time had yet to do in a consistent way [3]. Unfortunately, drawings by master artists such as Leonardo di Vinci did not receive notice by the physicians of the era, but the works of others would. In the early sixteenth century, Berengario da Carpi, professor at Bologna and Pavia, would produce drawings of the female uterus and would be the first to state clearly that the uterus consisted of one cavity [3, 6]. Two decades later Andreus Vesalius, professor of anatomy at Padua, with the aid of his illustrator, John of Calcar, would produce his epochal, “De Corporis Humani Fabrica.” In this work, Vesalius would reproduce an accurate description of the entire female genital tract including the ligaments of the uterus [6]. With this accomplishment, Vesalius and his disciples lifted the veil that had obscured the intricacies of the female genitourinary tract, ultimately helping physicians to better understand female pelvic floor anatomy.

## 3. Evolution of the Pessary

By the close of the sixteenth century, the management of uterine prolapse became more firmly rooted in the use of pessaries. Pessaries would evolve from lint balls or halved fruit soaked in vinegar to something closer to their modern form. This shift was largely due to the inventiveness of France's royal surgeon, Ambroise Paré. Paré devised oval shaped pessaries of brass and waxed cork. He attached thread to them to facilitate their removal, while others were to be worn with belts to help them remain in situ [8]. In the eighteenth century, Henrick van Deventer, who started his career as a goldsmith, made pessaries of various shapes and sizes out of waxed cork or wood, and metals such as silver and gold [9]. By the mid-nineteenth century, pessary use had become quite common. Yet, alternative methods of managing prolapse were still prescribed. These included the use of astringents such as tannin and alum; cold sitz baths, surf bathing, and sea-water douches; postural exercises; Brandt's “uterine gymnastics” which embodied anointing, massage, and manual replacement of the prolapsed parts; leeching; torsion of the uterus; attempts to produce fibrosis of the surrounding tissues by the introduction of gonorrheal exudates into the vagina or the deliberate induction of pelvic peritonitis [10]. 

Hugh Hodge of Philadelphia (who was concerned with ailments he believed to be caused by uterine retroversion) was a major proponent of pessary use. He shared the sentiments of many gynecologists in the United States and abroad when, in 1860, he proclaimed pessary use to be the “sine qua non” for the treatment of uterine displacements [11]. He put forth the following as the ideal qualities for a pessary: it should be made of incorruptible material, maintain the normal uterine position, allow for natural movement, be worn without pain, and not excite leucorrhea or menorrhagia [12]. The first of these, to be incorruptible, came to pass in 1844 when Charles Goodyear was granted U.S. patent no. 3,633 for the invention of vulcanized rubber [13]. Before then, pessaries had consisted of wax, wood, leather, glass, and metal. Now a material could be used that resisted decomposition. This ultimately led to the development of Hodge's eponymous lever pessary and was followed by an explosion in the number and variety of pessaries put to use by gynecologists. It was said in those years that fortunes were made by two groups of gynecologists: those who inserted pessaries and those who removed them (a bit reminiscent of vaginal mesh use today) [12]. 

However, not everyone in the profession was so keen on pessary use. In 1866, during his satirical presidential address to the New Hampshire State Medical Society, W. D. Buck commented, “The Transactions of the National Medical Association for 1864 has figured one hundred and twenty-three different kinds of pessaries, embracing every variety, from a simple plug to a patent threshing machine, which can only be worn with the largest hoops. They look like the drawings of turbine water-wheels, or a leaf from a work on entomology. Pessaries, I suppose, are sometimes useful, but there are more than there is any necessity for. I do think that this filling the vagina with such traps, making a Chinese toy-shop of it, is outrageous” [14]. Despite this sentiment, pessaries would remain popular throughout the eighteen hundreds. However, with the discoveries in asepsis by Lister and anesthesia by Morton, paired with advances in suture materials and surgical instruments, surgery would soon replace the pessary as the predominant method of treating uterine prolapse. 

## 4. The Rise of Surgery

The surgical management of uterine prolapse has been recorded as far back as the second century C.E. Soranus advised, “cutting off the black part,” when the prolapsed uterus became gangrenous [4]. Similarly, Berengario claimed he witnessed his father, a surgeon, remove a prolapsed uterus by scalpel asserting that not only had the patient survived, but also she was able to resume coitus. He later claimed to have achieved the same outcome using strong twine as an ecraseur [3]. Later, the prominent seventeenth century Dutch gynecologist Hendrik van Roonhuyse reported a case in which he extirpated a prolapsed uterus after multiple attempts by other caregivers had failed to adequately restore the organ (previously placed pessaries made of cork and wax had led to ulcerations, pain, foul discharge, putrefaction, and fever). The patient was reported to have survived, but van Roonhuyse provided no details of his surgical technique or of an anesthetic used, if any [3]. In these early reports it remains unclear whether “hysterectomy” meant removal of the cervix, the cervix and a portion of the uterus, or the uterus in total. 

During the mid to late 19th century, opening the peritoneum for any indication remained a risky endeavor and was largely reserved for cases of presumed gynecologic malignancy [15]. Consequently, surgical attempts to treat uterine prolapse consisted of efforts such as narrowing the vaginal vault (by colporrhaphy or the application of cautery or astringents), performing a perineorrhaphy or infibulation, or offering cervical amputation [10]. However, as the 19th century progressed, notable advances would take place. In 1877, the Frenchman LeFort—influenced by the works of German gynecologists such as Hegar, Simon, and Spiegelberg, who had the idea of occluding the vaginal introitus to restrain uterine prolapse—described the principle of partial colpocleisis, the operation that has borne his name since [16]. In 1886, Olshausen reported performing a laparotomy solely for the purpose of uterine ventrofixation [17]. In 1899, Watkins and Wertheim separately reported on the use of uterine interposition to treat uterine prolapse [12]. Although, by the end of the nineteenth century there were several treatments for uterine prolapse, the ability to achieve durable repairs remained elusive due to a limited understanding of female pelvic floor anatomy. 

## 5. Mechanisms of Uterine Support 

In 1895, while practicing in Berlin, Alwin Mackenrodt published his comprehensive, and accurate, description of the female pelvic floor connective tissue. In regard to what have become known as the Cardinal or Mackenrodt ligaments he remarked: “This whole ligamentous apparatus appears so excellent and extensive that it is quite surprising that it has not been recognized previously” [12]. Shortly thereafter, Fothergill, building upon the work of his senior colleague, the prominent Manchester obstetrician gynecologist Archibald Donald, recognized the importance of the Cardinal ligaments to uterine support and perfected what became known as the Manchester-Fothergill surgery. Fothergill's procedure involved dissecting the bladder off the lower uterine segment followed by plication of the parametrial and paravaginal tissue at the anterior aspect of the cervix, thus effectively shortening the uterine supports. He would combine the aforementioned steps with an anterior and posterior colporrhaphy and perineoplasty to keep recurrence in check [12, 18] and later would advocate cervical truncation as part of the surgery [19]. Fothergill would become a vociferous proponent of the belief that the parametrial (and paravaginal) fascia was the key structure to maintaining uterine support [20]. Referring to Peter Thompson's research on the comparative morphology of the levator ani muscles in tailed apes and man [21], he considered the levator ani muscles withered muscle bodies no longer required to carry out their original function (tail movement) and therefore deemed them inadequate supports for the uterine body. He remarked, “Injuries to the perineum and levator ani doubtless straighten and widen the road from the pelvic cavity to the exterior. But if the organs remain firmly attached above, no mere enlargement of the opening below will make them come down.” To bolster his thesis, Fothergill was fond of noting, “The true supports of the uterus can be seen at vaginal hysterectomy… Let him incise… round the cervix, and… freely divide the posterior attachments...Next let the operator deliver the fundus… this affords another proof that the broad and round ligaments have no value as suspenders… the uterus still remains fixed by the tissue known as the parametrium, and by this alone. Until this is divided… the organ is… as completely supported as before an incision was made” [20].

In 1934, Bonney published, “The Principles that Should Underlie All Operations for Prolapse.” Using basic analogies such as an in-turned finger of a rubber glove and the securing of stove piping in a metal box, Bonney was able to convey the manner in which the pelvic viscera are supported [22]. These concepts would later be refined by DeLancey and described as levels of fascial supports: level I: proximal suspension; level II: lateral attachments; level III: distal fusion [23].

In 1936, Mengert, inspired by 1858 cadaveric data from Legendre and Bastien, published a simple but influential study in which cadaveric uteri were subjected to traction with a 1 kg weight while structures attached to the uterus were severed in various sequences. The uterine descent observed after incising the parametrial tissues reinforced Mackenrodt's anatomic research and Fothergill's clinical observation, suggesting the parametrial and paravaginal tissues (i.e., cardinal and uterosacral ligaments) were the primary support structures for the uterus [24].

However, Mackenrodt and Fothergill were not lone voices. In 1907, a gynecologist, Josef Halban, and anatomist, Julius Tandler, professors at the famed Vienna Medical School [25], published Anatomie und Atiologie der Genitalprolapse beim Weibe [26]. Their thesis on uterine support was quite contradictory to Mackenrodt and Fothergill's. Halban and Tandler maintained that the pelvic fascia was like a spider's web, able to bear the proper weight of the spider, but incapable of supporting a greater, abnormal burden [27]. Thus, it was the levator ani muscles that were essential to maintaining uterine support. Like Fothergill, they too turned to the comparative anatomic work of Peter Thompson but drew a different conclusion. Consistent with a prime tenet of the Vienna School, form follows function [25], Halban and Tandler viewed the functional adaptation of the levator ani muscles from their tail wagging purpose to that of maintaining pelvic floor support as evidence they were not superfluous muscle bodies (otherwise they would have regressed with the tail), but significant [28]. Others who were sympathetic to Halban and Tandler's thesis would point to the observation of large prolapses in patients with mal-developed pelvic floor muscles from spina bifida, to the work of Goff, who asserted that the “fascia” described in vaginal plastic procedures was the “loosely arranged areolar type,” as well as the work of Berglas and Rubin, who demonstrated the complete absence of ligamentous material in the endopelvic fascia [27, 29, 30]. In time, pelvic floor surgeons would recognize the importance of both structures [31] influencing new approaches to repair uterine prolapse.

## 6. Vaginal Hysterectomy and Vault Prolapse

Vaginal hysterectomy was first performed and developed in attempts to treat cervical and uterine malignancies [15]. The first vaginal hysterectomy for uterine prolapse was reported by Choppin, of New Orleans, in 1861. The surgery was conducted under chloroform and the removal of the uterus, after it was dissected away from the bladder and rectum, was excised using “Chassaignac's Ecraseur.” A little more than a month after the surgery, Choppin presented the patient to the class of the New Orleans School of Medicine, the patient holding the specimen in hand [32]. Choppin's success was a rarity. However, as the new century arrived this fact would change. By 1915, Mayo would publish his technique for vaginal hysterectomy [33], as would Bissell, in 1918, coupling his technique of vaginal hysterectomy with an anterior and posterior colporrhaphy [34]. The rapid rise of surgery for the correction of uterine prolapse in the early twentieth century left one American gynecologist to write in 1923, “Gynecology has become so predominantly a surgical specialty… the young gynecologist of today frequently has no conception of what the pessary is meant to do and he is apt to be even irritated at the suggestion that such an implement should be accorded at least a modest position in his armamentarium” [35]. In the spring of 1937, at the sixty-second Annual Meeting of the American Gynecological Society, Baer and his colleagues reported on the type of operations performed for uterine prolapse in 1928 compared to those performed in 1937. They noted that by the latter date vaginal hysterectomy had become the predominant operation, replacing interposition [36]. Modifying the surgical methods established by Mayo, and others, McCall, in 1957, published his technique of obliterating the cul-de-sac of Douglas to cure an enterocele and prevent subsequent vault prolapse [37]. By the mid-twentieth century, vaginal vault prolapse had become a recognized sequela of hysterectomy. Thus, in 1965, Symmonds and Sheldon were able to report on the number of posthysterectomy vaginal vault prolapse cases they had observed at the Mayo Clinic [38]. 

Surgical attempts to correct posthysterectomy vault prolapse were made as early as the nineteen twenties. In 1927, Miller described a technique to reduce vault prolapse that amounted to a bilateral, transperitoneal iliococcygeus suspension (or, depending upon the actual depth of suture placement, a bilateral sacrospinous fixation) [39]. Others would follow with modifications of established procedures such as ventrofixation [40], with or without the use of a biograft [41, 42]. However, it was Arthure and Savage from Charing Cross Hospital in London who would make the most lasting impact on the repair of apical defects. They recognized that vault prolapse could occur after abdominal or vaginal hysterectomy, total or subtotal: hysterectomy alone would not cure uterine prolapse. They analyzed the surgical techniques used at the time and noted the faults of each. In 1957, they published their surgical technique of sacral hysteropexy believing it to be a better anatomic repair that would prove to have superior durability and less risk of enterocele formation. The description they provided, save the use of a graft, is nearly identical to the abdominal sacrocolpopexy performed today (they even noted the importance of keeping the repair tension free while using the sacral promontory as a fixation point) [43]. 

Long before the sacral promontory had been considered a fixation point for correcting apical prolapse, Zweifel of Germany, in 1892, commented on his attempts to correct uterovaginal prolapse by using silkworm sutures to unilaterlly affix the upper vagina to the sacrotuberous ligament

 [44]. The use of the sacrotuberous ligament to anchor vault prolapse was not attempted again until another German, J. Amreich, in the 1950s, reported on his experience using a transgluteal (Amreich I) and transvaginal (Amreich II) approach to a vaginal-sacrotuberal fixation [44]. Two other German gynecologists, Sederl and Richter, avoided the difficult-to-access sacrotuberous ligament in favor of the sacrospinous ligament, while attempting to repair vault prolapse transvaginally [45, 46]. Richter's operative success popularized his technique across Europe and also stimulated the interest of two American gynecologists, Randall and Nichols. In 1971, Randall and Nichols reported the surgical outcomes of 18 patients who underwent transvaginal sacrospinous fixation for vault prolapse performed over the previous four years. They found the operation restored the normal vaginal depth and felt it to be an effective operation in women with vault prolapse and in those who were found to have insufficient uterosacral or cardinal ligament strength at the time of vaginal hysterectomy [47].

Since Randall and Nichols' 1971 publication, the procedure has changed little [48, 49]. The most notable modifications have been related to instrumentation: the introduction of the Miya Hook [50], the Shutt needle driver [51], and the Laurus needle driver, presently known as the Capio (Boston Scientific, Natick, MA) [52]. Other reported surgical approaches to correct vault and advanced uterine prolapse include the iliococcygeus fixation (first described by Inmon) [53], endopelvic fascia fixation [54, 55], coccygeous muscle fixation [56], high uterosacral ligament suspension [57, 58], and levator myorrhaphy [59]. Thus, upon exiting the twentieth century, it had been the effort and ingenuity of a multitude of accomplished surgeons, attempting to prevent and correct vaginal vault prolapse, which led to many of the surgical techniques presently used to correct advanced apical prolapse. Notably, of the surgeries established in the nineteenth century, only the LeFort colpocleisis has endured.

## 7. Quantifying Prolapse

In October 1995, the International Continence Society formally adopted the document that would introduce the Pelvic Organ Prolapse Quantification (POP-Q) to the larger gynecology community. This document, three years in the making, and validated in six centers in Europe and the United States, would replace Baden-Walker and other descriptive measures as the means to objectively report findings of pelvic organ prolapse [60]. Subsequently, the POP-Q has become the standard means by which to report pelvic organ prolapse in the international literature and has been increasingly embraced by physicians in their clinical practices [61]. However, the POP-Q is not without its potential confounders [62, 63]. Thus, both clinicians and clinical investigators have turned to the various imaging modalities that allow for in situ evaluation of the female pelvic organs and their supporting structures. 

Imaging pelvic floor anatomy can be traced back to Berglas and Rubin's method of levator myography in which they injected radio-opaque dye into the levator ani muscles, vagina, and endocervix, revealing by X-ray that the vagina did not rest at a steep incline, but rather lie almost horizontal, parallel to the levator plate [64]. Since that time, both magnetic resonance imaging (MRI) and sonography have advanced notably to better visualize the pelvic floor. Hedvig Hricak first described female pelvic anatomy by MRI in 1983 [65]; however, he was most concerned with its ability to differentiate benign versus malignant conditions involving the pelvic organs [66]. Yang and colleagues, in 1991, would introduce dynamic MRI. This would allow MR images to be taken during valsalva [67]. Further, 2D and 3D MRI has been used in research studies to evaluate levator ani status in women with and without pelvic floor disorders [68, 69]. 

The most recent advances in MRI technology, such as HASTE (half-Fourier-acquisition single shot turbo spin echo technology), FISP (fast imaging with steady-state free precession), and TSE (turbo spin-echo), allow for the fast acquisition of images simultaneously in all three compartments (anterior, central, and posterior), making MRI a valuable option to aid in the evaluation of pelvic floor disorders, including prolapse. Already, MRI is replacing fluoroscopy as the means to perform defecography studies in some institutions, and it continues to be evaluated in research protocols investigating its potential role in the clinical evaluation of pelvic organ prolapse [70]. 

Although MRI is fascinating technology, it has its flaws: exams are performed in the supine position, it does not allow for patient biofeedback during imaging, it may not be tolerated well by some patients, and it is costly. As an alternative, sonography which has been utilized to aid in evaluating the urogynecologic patient since the mid-1980s has the advantage of lower cost, relative ease of use, minimal patient discomfort, shorter study durations, and wide availability [71, 72]. The advent of 3D/4D sonographic imaging has improved the clinical utility of pelvic floor sonography, and the transperineal/translabial approach has made it more patient friendly. Dietz and colleagues have reported 3D/4D sonography to be more accurate than physical exam in detecting levator muscle injuries and that sonographic injuries to the levator muscles are associated with pelvic organ prolapse, including apical prolapse [73, 74]. Sonography has also been used to image vaginal mesh implants, as it is able to readily detect mesh size and position (as opposed to MRI or CT) [75]. 

## 8. Apical Prolapse Surgery in the 21st Century 

Two major shifts have occurred in the surgical management of apical prolapse in current practice: the introduction of vaginal mesh and that of advanced endoscopic surgery.

Graft use in pelvic reconstructive surgery can be traced back to the early 1900s [76]. In 1955, Moore and colleagues reported the use of tantalum mesh in the repair of cystoceles [77]. The concept that pelvic organ prolapse is a type of hernia, comparable to other fascial defects, made attractive the idea of replacing weakened fascia of the pelvic floor with a more reliable biologic or synthetic material. Over the intervening years, a number of auto-, allo-, and xenografts have been used with this intent in pelvic floor repairs. However, the success general surgeons achieved using polypropylene mesh in the correction of incisional hernias significantly influenced the use of this mesh by pelvic floor surgeons (type I monofilament, macroporous polypropylene mesh becoming the standard) [78]. Additionally, the success of the tension-free transvaginal tape (TVT) mid-urethral sling with its facility of use, clinical effectiveness, and marketability as an all-inclusive “kit” demonstrated the potential for mesh to improve surgical outcomes and opened a market in women's health care medical device manufacturers could exploit. In 2001, Petros introduced the infracoccygeal sacropexy (Intravaginal Slingplasty Tunneler, Tyco, USA) as a novel means to transvaginally correct vault prolapse using polypropylene mesh [79]. Petros' mesh was multifilament and complications due to perirectal abscesses and fistula formation led to its removal from the market. However, since that time, a steady stream of mesh “kits” have been engineered by medical device makers and have made their way into the hands of many pelvic floor surgeons for the purpose of correcting apical and other forms of prolapse. Yet controversy has and continues to surround the use of vaginal mesh particularly as its acceptance in clinical use has outpaced the development of well-designed clinical trials [80]. In 2006, the French Health Authorities (HAS) reported that mesh for transvaginal repair of pelvic organ prolapse should be limited to clinical research [81]. In 2008, the US Food and Drug Administration (FDA) issued a warning regarding the use of mesh for prolapse and incontinence repair [82], repeating that warning in 2011, although narrowing it to vaginal mesh used to correct pelvic organ prolapse (not for anti-incontinence procedures or when used abdominally) [83]. These warnings stemmed from concerns over mesh erosion through the vagina, pain, infection, bleeding, dyspareunia, organ perforation, and urinary problems. While many of these complications are common to all pelvic floor repairs, mesh erosion and some types of organ perforation are surely unique to mesh and the trocars used for its placement. Presently, with respect to apical prolapse, no published, well-designed, randomized controlled trials have established the superiority of vaginal mesh over native tissue repairs [84]. This is beginning to change with respect to the anterior compartment [85]. 

What the future holds for vaginal mesh in pelvic organ prolapse repairs is uncertain. Nevertheless, while biomaterials improve and the subspecialty weighs the appropriate indications for their use, advances in endoscopic repairs for apical prolapse surge forth.

It has been the efforts of many physicians from the global scientific community that have brought forth the modern state of laparoscopy in gynecologic surgery. The pioneering works of Georg Kelling, Hans Christian Jacobaeus, John C. Ruddock, Janos Verees, and Kurt Semm all deserve further mention; however, a discussion of their contributions is beyond the scope of this paper [86–88]. 

The recent advances in endoscopic technology have been remarkable, and they have allowed urogynecologists to make endoscopic surgery a primary tool in their surgical armamentarium. It has been of great benefit to patients that what many believe to be the most durable apical prolapse repair, abdominal sacrocolpopexy, is achievable via minimally invasive approaches [89]. Presently, a debate exists regarding what method of sacrocolpopexy (straight sticks versus robotic assisted) should become the predominant technique taught and performed by urogynecologist in light of differences in cost, patient safety, surgeon training, and surgical outcomes. Well-designed studies have started to shed light on this issue [90, 91], but it is a conversation that is only beginning. And yet, in the shadow of that debate gynecologists are already reporting their early experiences with single incision laparoscopic surgery (SILS, or LESS—laparoendoscopic single-site surgery) and natural orifice transluminal endoscopic surgery (NOTES) [92, 93]. Whether these new surgical approaches will be amenable to performing safe and timely apical and other prolapse repairs remains to be seen. Nevertheless, a SILS sacrocolpopexy has been reported [94]. 

## 9. Conclusion

Uterine prolapse is an age-old condition the treatment of which has evolved over thousands of years. It is a condition from which many women have suffered and that many physicians have attempted to treat. The slow historical progress of the field and the challenges that we face today in treating uterine prolapse reflect the very intricacies of this disorder that fascinate and inspire us. Today, not only do urogynecologists reap the benefits gleaned from the developments over the ages, but also from the advances in modern technology. We are now positioned to more effectively evaluate and treat this condition and to enhance our understanding of its causes through the pursuit of novel research. 
